# Supplementary material for: Intrinsic anharmonic localization in thermoelectric PbSe
Source: Nat Commun. 2019 Apr 26;10:1928. doi: 10.1038/s41467-019-09921-4 (PMC6486597; doi:10.1038/s41467-019-09921-4)
Supplement: Supplementary file 1 — Supplementary Information [file 41467_2019_9921_MOESM1_ESM.pdf]

Supplementary Information

Intrinsic anharmonic localization in thermoelectric PbSe

Manley et al.

Supplementary Figures

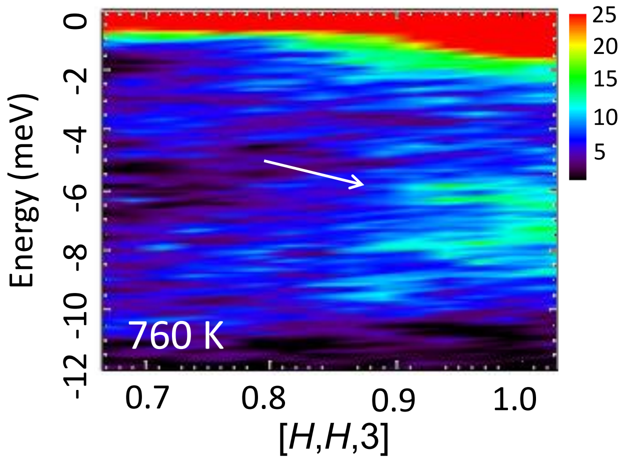

Supplementary Figure 1. Time-of-flight cold neutron scattering for annihilated excitations. Although less sharp because of slightly lower resolution, the splitting off of a small flat feature is apparent and consistent with the positive neutron energy transfer side (Fig. 2).

a

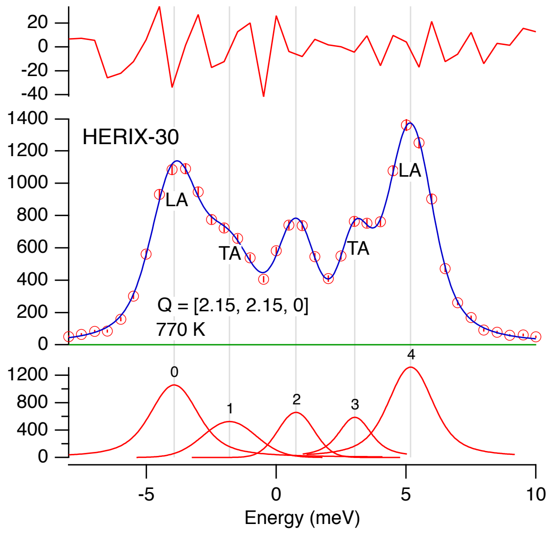

Peak 0	Type: Voigt			
	Location =	-3.9317	+/-	0.074849
	Height =	1058.1	+/-	2323.5
	Area =	3099.6.4	+/-	282.11
	FWHM =	2.1784		
	Gauss Width =	1.50	+/-	0
	Lorenz Width =	1.1393	+/-	0.051797
Peak 4	Type: Voigt			
	Location =	5.1501	+/-	0.019894
	Height =	1351.8	+/-	214.75
	Area =	3726	+/-	100.25
	FWHM =	2.1068		
	Gauss Width =	1.50	+/-	0
	Lorenz Width =	1.0308	+/-	0.027765

b

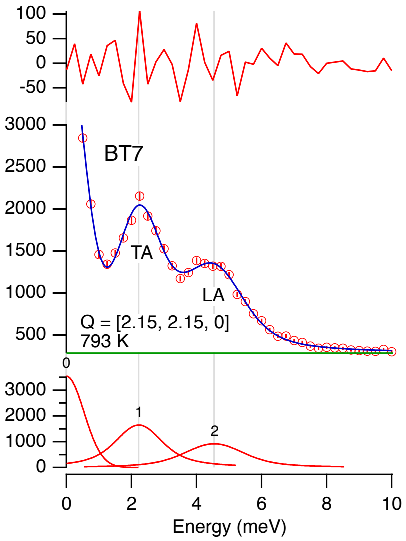

Peak 2	Type: Voigt			
	Location =	4.5407	+/-	0.042296
	Height =	919.94	+/-	449.89
	Area =	2676.1	+/-	174.71
	FWHM =	2.185		
	Gauss Width =	1.50	+/-	0
	Lorenz Width =	1.1653	+/-	0.064828

Supplementary Figure 2. Peak fitting procedure for the x-ray and neutron measurements. **a**, Fits to the HERIX (x-ray) data for **Q** = [2.15,2.15,0] at 770 K. A Voigt function was used to fit the phonon peaks. This function is defined as the convolution of a Gaussian distribution and Lorentz distribution. The Lorentz distribution is a good approximation to the lifetime broadening of a phonon, whereas a Gaussian distribution is a good approximation to the instrument resolution function. Therefore, we fix the Gaussian width to the instrument resolution of 1.5 meV and let the fit determine the Lorentzian width from phonon lifetime broadening. The raw data shown is offset slightly in energy, correcting for this the LA peak position is 4.54 meV. **b**, Same fit procedure applied to the BT7 (neutron) data for **Q** = [2.15,2.15,0] at 793 K, gives an LA peak position also at 4.54 meV and a similar linewidth.

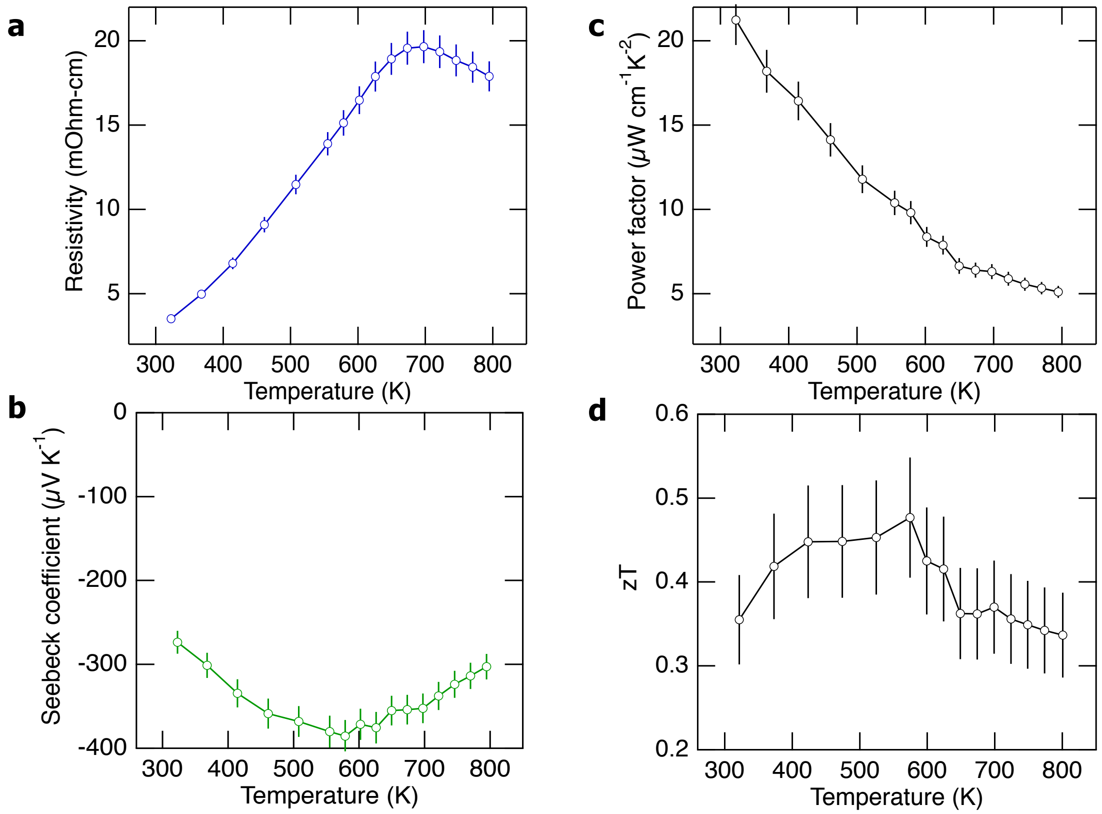

Supplementary Figure 3. Thermoelectric properties of PbSe single crystal. a, Electrical resistivity; **b**, Seebeck coefficient; **c**, Power factor; **d**, Thermoelectric figure of merit, $zT = \frac{\sigma S^2 T}{\kappa}$, where S is the Seebeck coefficient, σ is the electrical conductivity, T is temperature, and κ is the thermal conductivity (Power factor = σS^2). Thermal diffusivity results from Fig. 5 were used to calculate thermal conductivity using: $\kappa = \alpha \rho C_P$ in which α is thermal diffusivity, ρ is density and C_P is the specific heat from Ref. [1]. The error bars are based on systematic errors determined by the manufacturer by testing a reference material over a long period of time. These are combined to determine a composite error on zT as described in Ref. [2].

Supplementary references

1. Pashinkin, A. S., Mikhailova, M. S., Malkova, A. S. & Fedorov, V. A. Heat capacity and thermodynamic properties of lead selenide and lead telluride. *Inorganic Mater.* **45**, 1226-1229 (2009).
2. Wang, H. et al. International Round-Robin Study on Thermoelectric Transport Properties of n-type Half-Heusler from 300 K to 773 K. *Journal of Electronic Materials* **44** (11), 4482-4491 (2015).
